# Long-Term Oncologic Outcomes of Off-Clamp Robotic Partial Nephrectomy for Cystic Renal Tumors: A Propensity Score Matched-Pair Comparison of Cystic versus Pure Clear Cell Carcinoma

**DOI:** 10.3390/curroncol31060227

**Published:** 2024-05-27

**Authors:** Mariaconsiglia Ferriero, Alberto Ragusa, Riccardo Mastroianni, Gabriele Tuderti, Manuela Costantini, Umberto Anceschi, Leonardo Misuraca, Aldo Brassetti, Salvatore Guaglianone, Alfredo Maria Bove, Costantino Leonardo, Michele Gallucci, Rocco Papalia, Giuseppe Simone

**Affiliations:** 1 Department of Urology, IRCCS “Regina Elena” National Cancer Institute, 00144 Rome, Italy; riccardo.mastroianni@ifo.it (R.M.); gabriele.tuderti@ifo.it (G.T.); manuela.costantini@ifo.it (M.C.); umberto.anceschi@ifo.it (U.A.); leonardo.misuraca@ifo.it (L.M.); aldo.brassetti@ifo.it (A.B.); salvatore.guaglianone@ifo.it (S.G.); alfredo.bove@ifo.it (A.M.B.); costantino.leonardo@ifo.it (C.L.); michele.gallucci@gmail.com (M.G.); giuseppe.simone@ifo.it (G.S.); 2 Department of Urology, Fondazione Policlinico Universitario Campus Bio-Medico, 00128 Rome, Italy; alberto.ragusa@unicampus.it (A.R.); rocco.papalia@policlinicocampus.it (R.P.)

**Keywords:** cystic renal tumors, robotic surgery, nephron-sparing surgery, off-clamp partial nephrectomy, oncological outcomes

## Abstract

Few data are available on survival outcomes of partial nephrectomy performed for cystic renal tumors. We present the first long-term oncological outcomes of cystic (cystRCC) versus pure clear cell renal cell carcinoma (ccRCC) in a propensity score-matched (PSM) analysis. Our “renal cancer” prospectively maintained database was queried for “cystRCC” or “ccRCC” and “off-clamp robotic partial nephrectomy” (off-C RPN). The two groups were compared for age, gender, tumor size, pT stage, and Fuhrman grade. A 1:3 PSM analysis was applied to reduce covariate imbalance to <10% and two homogeneous populations were generated. Student t- and Chi-square tests were used for continuous and categorical variables, respectively. Ten-year oncological outcomes were compared between the two cohorts using log-rank test. Univariable Cox regression analysis was used to identify predictors of disease progression after RPN. Out of 859 off-C RPNs included, 85 cases were cystRCC and 774 were ccRCC at histologic evaluation. After applying the PSM analysis, two cohorts were selected, including 64 cystRCC and 170 ccRCC. Comparable 10-year cancer-specific survival probability (95.3% versus 100%, *p* = 0.146) was found between the two cohorts. Conversely, 10-year disease-free survival probability (DFS) was less favorable for pure ccRCC than cystRCC (66.69% versus 90.1%, *p* = 0.035). At univariable regression analysis, ccRCC histology was the only independent predictor of DFS probability (HR 2.96 95% CI 1.03–8.47, *p* = 0.044). At the 10-year evaluation, cystRCC showed favorable oncological outcomes after off-C RPN. Pure clear cell variant histology displayed a higher rate of disease recurrence than cystic lesions.

## 1. Introduction

Renal cell carcinoma (RCC) represents about 3% of all forms of cancer and about 90% of malignant lesions of the kidney. The three primary types of RCC are clear cell RCC (ccRCC), papillary RCC (pRCC), and chromophobe RCC (chRCC) (constituting up to 70–85%, 10–15%, and 4–5% of cases, respectively). A minority of solid kidney neoplasms, ranging around 2.5–18%, is cystic renal cell carcinoma (cystRCC) [1].

A recent update to Bosniak classification subdivided cystic lesions into five categories based on CT or MRI diagnostic criteria. Renal cysts rated as IIF, III, and IV are malignant approximately in 0–38%, 50%, and 100% of surgically treated cases, respectively [2]. Being considered a good predictor of malignancy, the classification suggests the clinical management for follow-up and treatment of cystic lesions. 

The most common histological subtype for Bosniak III cysts is ccRCC, with pseudocystic changes and low malignant potential [1]. Other histological features, such as tubulocystic RCC or cystic nephroma/mixed epithelial and stromal tumors, also known as renal epithelial and stromal tumors (REST), appear as Bosniak type II/IV. Acquired cystic disease-associated RCC is associated with end-stage renal disease (ESRD). According to the WHO 2022 classification, multilocular cystic renal neoplasm (mcRCC) of low malignant potential is a new subtype of ccRCC, since multiple publications reported no recurrence or metastasis in patients with mcRCC [1,3]. Tubulocystic RCC, acquired cystic disease-associated RCC, and eosinophilic cystic RCC were considered other independent tumor entities [4].

These variant histologies have a low malignant potential, and nephron-sparing surgery is the standard approach when it is technically feasible [1]. Therefore, a minimally invasive partial nephrectomy is achievable according to surgeon skills and has been widely reported [5,6,7,8,9]. There are few studies showing oncological outcomes of cystRCC compared to “pure” ccRCC, defined as a tumor without any cystic differentiation at pathologic examination. Additionally, there are limited data available with a long-term follow-up [9,10]. All series reported in the literature, except the one by Novara et al. [8], are single-center, small, and retrospective experiences [7,9,10]. As a result, comprehensive and conclusive information concerning the behavior of cystRCC versus solid pure ccRCCs, as well as their long-term survival outcomes, remains relatively insufficient.

We report 10-year oncological results of cystRCC compared to pure ccRCC treated with robotic partial nephrectomy in a propensity score-matched (PSM) analysis.

## 2. Materials and Methods

### 2.1. Study Design and Patient Selection

**Figure 1:**
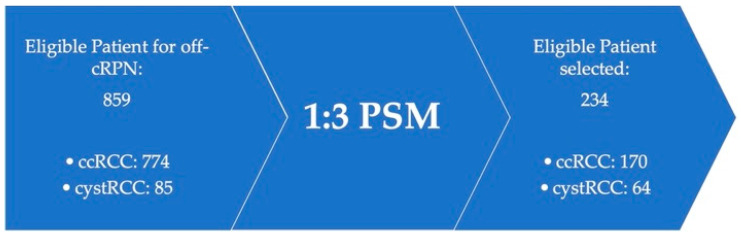
Flowchart diagram of the study before and after 1:3 PSM analysis. Off-c RPN: off-clamp robotic partial nephrectomy; ccRCC: clear cell renal cell carcinoma; cystRCC: cystic renal cell carcinoma; PSM: propensity score matched.

Between January 2013 and January 2022, our internal IRB approved and prospectively maintained “renal cancer” database was queried for “off-clamp robotic partial nephrectomy” (off-C RPN) and “cystic” or “pure clear cell” variant histology at pathologic examination. A total of 859 patients who received robotic partial nephrectomy were included in the study, with 774 ccRCC and 85 cystRCC. After 1:3 PSM analysis, 170 patients with ccRCC, and 64 patients with cystRCC were selected (Figure 1). Exclusion criteria were gross hematuria or tumor infiltration of the urinary tract observed at conventional imaging. After obtaining informed written consent before the procedure, peri-operative data were collected.

Baseline imaging was reviewed for all cases to properly assess tumor characteristics.

### 2.2. Surgical Technique

All patients received RPN with the pure enucleation technique, regardless of the cystic or solid macroscopic aspect of renal masses; enucleo-resection was performed only when necessary, sometimes to manage complex cystic lesions in order to avoid rupture. Off-clamp has always been the standard approach for RPN in our institution. All cases were performed by two skilled surgeons.

A 30-degree scope was utilized for visualization, and a total of two robotic and two laparoscopic ports were placed. The assistant surgeon’s two 12 mm ports were positioned between the camera and the robotic ports, forming a “U” shape targeting the tumor. The colon was medialized, and the Gerota fascia was carefully opened. Subsequently, dissection proceeded through renal capsula to discover the tumor margins and to start enucleation. Monopolar scissors were used to mark the tumor margins circumferentially. The pneumoperitoneum pressure was increased to 20 mmHg, and the enucleation plane was gradually developed employing blunt dissection. To ensure a bloodless field visualization, two suction devices were employed concurrently, which allowed for both irrigation and suction. The specimen, referring to the excised tissue, was placed in an endocatch bag and extracted. The resection bed was carefully examined, and any small arterial feeders that were not initially controlled during the development of the enucleation plane were managed using monopolar pinpoint coagulation.

When needed, a hemostatic agent such as Tabotamp or absorbable fibrillar was applied to improve hemostasis and fill the parenchymal defect. A sliding-clip renography was performed in case of suspicious opening of the caliceal system. The renal capsule was sealed to reduce the risk of fluid leakage into the peritoneal space and a drain was left in place, which was usually removed on the first post-operative day.

Moreover, we previously reported the feasibility of off-c PN even in challenging cases, such as large tumors (cT2 renal masses) [11,12], purely hilar lesions, and totally endophytic lesions, with the help of Indocyanine Green technology when indicated [13,14,15].

### 2.3. Follow-Up Schedule

Follow-up visits encompassed complete biochemical blood tests (including serum creatinine, electrolyte levels, urea nitrogen and uric acid), an accurate physical examination, an abdominal ultrasonography and a chest X-ray or CT scan alternatively. Follow up was scheduled every six months for the first two years and yearly thereafter.

### 2.4. Statistical Analysis

The main clinical features were compared between the two groups. A 1:3 PSM analysis was used to obtain two populations homogeneous for age, gender, tumor size, RENAL nephrometry score [16], pT stage, and Fuhrman grade to decrease covariate imbalance to <10%. Continuous data were presented as median and interquartile ranges (IQR). Mann–Whitney and Chi-square tests were employed to compare continuous and categorical variables, respectively. A two-sided *p*-value < 0.05 was considered statistically significant. Ten-year disease-free survival (DFS), cancer-specific survival (CSS), renal recurrence-free survival (RRFS, defined as the time to evidence of tumor recurrence in the kidney or perirenal field), and metastasis-free survival (MFS) were compared between the two study cohorts using the log-rank test. Age, gender, Fuhrman grade, pT stage, tumor size, necrosis, and variant histology were included in a univariable Cox regression analysis to find predictors of disease development following partial nephrectomy. Statistical analysis was performed using the Statistical Package for Social Science (SPSS) v. 24.0 (IBM, Somers, NY, USA).

## 3. Results

**Figure 2:**
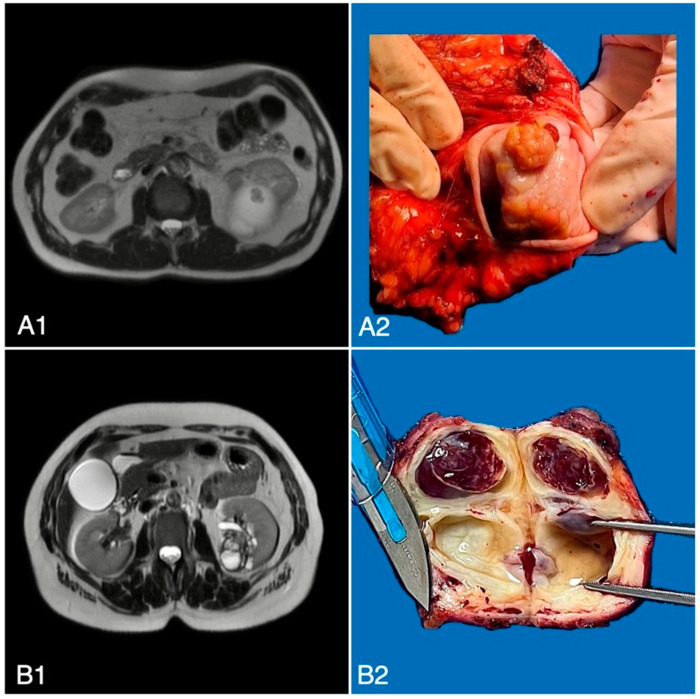
(**A1**) Bosniak IV lesion; (**A2**) cystic papillary renal cell carcinoma type 2 pT2a; (**B1**) Bosniak IV lesion; (**B2**) cystic clear cell renal cell carcinoma G2 pT1b.

All clinical and pathological features of the study cohorts are reported in Table 1. Overall, 85 cases were cystRCC and 774 were pure ccRCC at pathologic examination. Of the 85 cystRCC, 23 were pure cystic tumors and 62 were papillary or clear cell RCC mixed with cystic components. Figure 2 shows a radiologic and macroscopic feature of Bosniak IV cystic lesions, which were found to be cystic papillary type 2 and cystic clear cell RCC, respectively, at pathologic examination.

The two groups were not homogeneous for age, tumor size, RENAL score, pT stage, or Fuhrman grade (*p* < 0.001; *p* = 0.001; *p* = 0.014; *p* < 0.001; *p* < 0.001, respectively).

After applying the PSM analysis, two cohorts were selected, including 64 cystRCC and 170 pure ccRCC, which were homogeneous for all tested variables. The median follow-up was 46.5 months (IQR 21–80.75 months). Conversion to an open approach or from partial to radical nephrectomy never occurred in either cohort. The high-grade (3–5) Clavien complication rate was comparable between the ccRCC and cystRCC groups (2.2% versus 2.5%, *p* = 0.65 respectively).

After PSM analysis, the mean tumor size was 3.82 cm versus 3.85 cm for ccRCC and cystRCC, respectively (*p* = 0.65), and consequently, the most prevalent pathological tumor stage was T1a for both the solid and the cystic group (67.1% vs. 67.2%; *p* = 0.86). Fuhrman grade 2 was the most detected for both cohorts (66.3% vs. 50.6% for solid and cystic group, respectively; *p* < 0.001).

**Figure 3:**
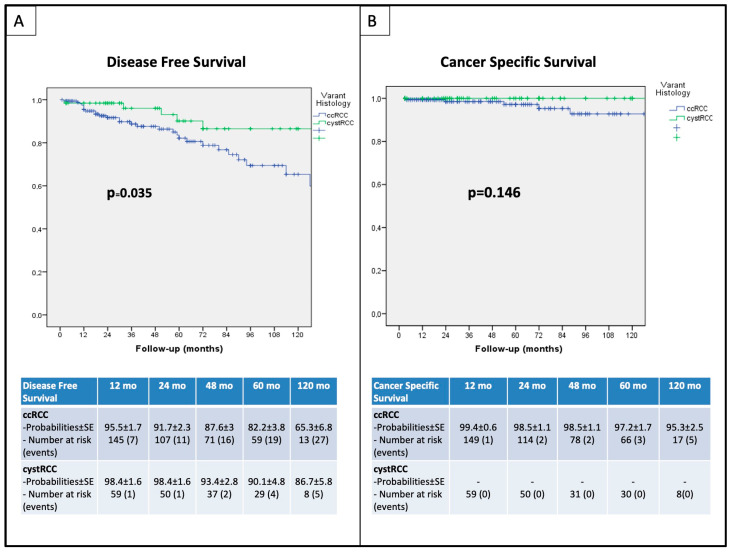
Kaplan–Meier survival analysis: (**A**) disease-free survival (DFS); (**B**) cancer-specific survival (CSS).

**Figure 4:**
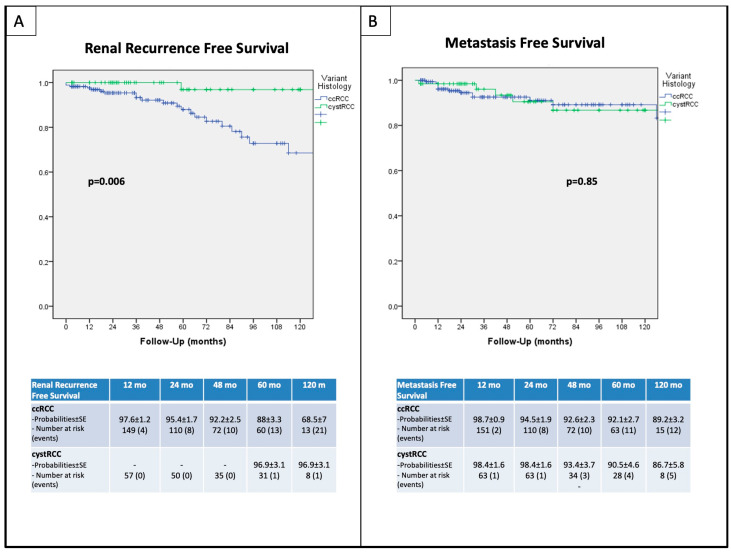
Kaplan–Meier survival analysis: (**A**) renal recurrence-free survival (RRFS); (**B**) metastasis-free survival (MFS).

Ten-year DFS, CSS, RRFS, and MFS were 66.69%, 95.3%, 68.5%, and 89.2% versus 90.1%, 100%, 96.9%, and 86.7% for pure ccRCC and cystRCC, respectively (log-rank *p* = 0.035, *p* = 0.146, *p* = 0.006, and *p* = 0.851, respectively). Kaplan–Meier curves have been reported in Figure 3 and Figure 4. At univariable regression analysis, pure ccRCC histology was the only independent predictor of DFS (HR 2.96 95% CI 1.031–8.478, *p* = 0.044).

## 4. Discussion

The indication of surgical treatment for renal cystic lesions arose from Bosniak classification [1,2]. If surveillance is a viable option for selected Bosniak III cases, a surgical approach should be performed in Bosniak IV patients [1]. Partial nephrectomy (PN) represents the standard treatment for renal masses whenever surgically feasible, including cystRCC [1].

Nevertheless, although most of the studies have focused on PN for renal masses in general, the feasibility and safety of this treatment for cystic lesions has been poorly investigated [7,8,9,10].

Our experience of partial nephrectomy was entirely built on the off-clamp technique, with excellent long-term functional and oncological outcomes [11,12,13,14,15]. Bertolo et al. reported in a PSM analysis comparable overall survival (OS, *p* = 0.1), recurrence (χ^2^ = 0.008, *p* = 0.9), or MFS (χ^2^ = 0.962, *p* = 0.3) between on-clamp and off-clamp RPN. Only one cancer-specific death occurred in the off-clamp group. Therefore, PN can be considered a safe technique regardless of the clamping approach [17].

The absence of positive surgical margins is one of the crucial trifecta endpoints for PN.

In a multicentric study, Ani et al. reported comparable 5-year DFS (90.9% versus 91.9%, log-rank *p* = 0.58) and OS (84.4% versus 88.6%, log-rank *p* = 0.58) in patients with or without positive surgical margins who received PN performed with any approach [18]. In our series, the positive surgical margin rate was comparable between ccRCC and cystRCC cohorts (1.2% vs. 0%, respectively, *p* = 0.38). However, at univariable regression analysis, only pure ccRCC histology was found to be predictive of DFS probabilities (HR 2.96 95% CI 1.031–8.478, *p* = 0.044).

An accurate resection avoiding rupture during enucleation of a cystic renal lesion is expected in order to prevent possible tumor seeding. However, there are no reliable data showing differences in DFS probabilities in the case of intraoperative cyst rupture. Pradere et al. reported a comparable estimated 5-year RFS between patients with versus without intraoperative cystic rupture (100% vs. 92.7%, respectively, *p* = 0.20) [19]. In the present study, a specific focus on cystic rupture is missing; however, we reported a more favorable 10-year oncological outcome of cystRCC versus pure ccRCC treated with off-c RPN. To the best of our knowledge, this is the first manuscript comparing two variant histologies of renal tumors without relying on cross-sectional imaging and providing such a long-term follow-up.

In a single-center retrospective experience comparing solid versus complex cystic renal lesions treated with robotic on-clamp PN, Raheem et al. reported at a median follow-up of 58 months, with a recurrence rate and cancer-specific mortality of 4.1% and 2%, respectively, for solid tumors, while cystic lesions displayed no recurrence rate and 100% OS probability. Five-year DFS, CSS, and OS probabilities were comparable between the two groups [9].

Similar oncological outcomes were found by Zennami et al. in a single-center retrospective study of robotic on-clamp PN for cystic and solid tumors, with comparable DFS, CSS, and OS (log-rank *p* = 0.18, *p* = 0.55, and *p* = 0.18, respectively) [10]. At 41-month follow-up, the recurrence rate (defined as tumor relapse in the operative field or the presence of lymph node or distant metastasis) was 3.7% in the solid group, while no recurrence for cystic tumors was detected (*p* = 0.368) [10]. These data, provided from retrospective and not homogeneous series, weakened the reliability of the studies.

In our experience of off-C RPN, selection bias was overcome using a PSM analysis, and an adequate follow-up strengthened our findings. At a median follow-up of 46.5 months (IQR 21–80.75), we reported favorable survival outcomes for both cohorts; specifically, comparable 5-year and 10-year CSS (97.2% and 95.3% versus 100%, respectively, *p* = 0.146), were found. Conversely, 5-year and 10-year DFS were less favorable for pure ccRCC than cystRCC (84.1% and 66.69% versus 90.1%, respectively, *p* = 0.035).

Specifically, comparable MFS probabilities between ccRCC and cystRCC (89.2% vs. 86.7%, respectively, *p* = 0.85) were found, while pure ccRCC displayed a poorer RRFS compared to cystRCC (68.5% vs. 96.9%, respectively, *p* = 0.006).

Cystic lesions, being approximately up to 18% of solid kidney neoplasms, exhibited discrepancies between radiological and pathological evaluations [1]. The updated Bosniak Classification of Cystic Renal Masses provides a framework for categorizing cystic lesions based on imaging characteristics [2]. In a large cohort of patients with radiographically confirmed cystic renal masses managed through active surveillance or intervention, Lee et al. revealed a notable discordance between radiographic and pathological designations. Over 80% of radiographically Bosniak cystic lesions were not described as “cystic” on pathology reports [20]. Since critical questions were raised about the reliability of imaging-based classifications in predicting pathological characteristics, we reported surgical and oncological outcomes of pathologic cystRCC treated with off-c RPN.

Moreover, in retrospective analysis exploring the 2019 version of the Bosniak Classification, Tse J.R. et al. underscored the challenge of risk stratification, specifically for class III and IV cystic masses. The prevalence of malignancy ranged from 56% to 61% for class III and 83% for class IV, with subclassifications demonstrating varying malignancy rates [21]. In view of the reported excellent outcomes of these patients, a surveillance approach could be an alternative to surgical treatment in Bosniak III cysts, avoiding the risk of overtreating 49% of the tumors that are lesions with a low malignant potential [1,22,23].

Interestingly, the use of machine-learning algorithms to predict ccRCC growth rate classes based on MRI or to identity malignant cystic lesions by a radiomic CT-based model could be useful for the individualized management of renal tumors [24,25].

The present study is not devoid of limitations. The single-center experience, the small sample size, and the low reproducibility of the off-clamp approach out of a tertiary referral center are considered the main drawbacks. Although our experience has shown excellent long-term results, as mentioned previously [11,12,13,14,15,17], we acknowledge that the reproducibility of this technique may vary across different institutions. Further prospective, multicenter studies would be useful to define the best management of cystic renal tumors.

## 5. Conclusions

Off-clamp RPN is a suitable treatment option for cystic renal tumors. At a 10-year evaluation, cystic variant histology showed favorable oncological outcomes, while pure ccRCC displayed a higher rate of renal recurrence than cystic tumors. These findings highlight the impact of histological subtypes on disease survival probability and emphasize the potential feasibility and safety of off-clamp RPN in managing cystic renal masses.

## Figures and Tables

**Table 1:** Clinical and pathologic data of the whole cohort and after the propensity score (PS)-matched analysis. SD: standard deviation; M: male; F: female; N: number.

	Whole Cohort		*p* Value *	1:3 PS Matched Cohort		*p* Value *
	ccRCC	cystRCC		ccRCC	cystRCC	
	(774)	(85)		(170)	(64)	
Age (years), mean (SD)	60.3 (12.4)	53 (15.3)	**<0.001**	56.52 (13.4)	55.4 (15)	0.57
Gender N (%)			0.283			0.42
M	500 (64.6)	60 (70.6)		110 (64.7)	45 (70.3)	
F	274 (35.4)	25 (29.4)		60 (35.3)	19 (29.7)	
ASA score 3						
(American Society of Anesthesiologist)	184 (23.8)	13 (15.3)	0.11	38 (21.7)	11 (18.6)	0.44
Tumor size (cm) Mean (SD)	3.7 (2)	4.4 (3.2)	**0.001**	3.82 (2)	3.85 (1.9)	0.65
RENAL score Median (range)	7 (6–9)	9 (8–10)	**0.014**	8 (6–9)	9 (8–10)	0.13
pT stage N (%)			**<0.001**			0.86
1a	488 (63)	54 (63.5)		114 (67.1)	43 (67.2)	
1b	215 (27.8)	17 (20)		40 (23.5)	13 (20.3)	
2a	28 (3.6)	9 (10.6)		11 (6.5)	5 (7.8)	
2b	41 (5.4)	3 (3.5)		5 (2.9)	3 (4.7)	
3a	1 (0.1)	2 (2.4)		-	-	
Fuhrman grade N (%)			**<0.001**			
1	49 (6.3)	27 (31.8)		35 (20.6)	17 (26.6)	
2	513 (66.3)	43 (50.6)		111 (65.3)	39 (60.9)	
3	201 (26)	9 (10.6)		24 (14.1)	8 (12.5)	
4	11 (1.4)	0		-	-	
Necrosis N (%)	61 (7.9)	2 (2.4)	0.077	16 (9.4)	2 (3.1)	0.17
Positive Surgical margins N (%)	16 (2.1)	1 (1.2)	0.576	2 (1.2)	0	0.38

* Student t- and Chi-square tests were used for continuous and categorical variables, respectively.

## Data Availability

Data presented in this study is available on request from the corresponding author.

## References

[1] Ljungberg B., Albiges L., Bedke J., Bex A., Capitanio U., Giles R.H., Hora M., Klatte T., Marconi L., Powles T. (2023). EAU Guidelines on Renal Cell Carcinoma. Edn. Presented at the EAU Annual Congress Milan 2023.

[2] Silverman S.G., Pedrosa I., Ellis J.H., Hindman N.M., Schieda N., Smith A.D., Remer E.M., Shinagare A.B., Curci N.E., Raman S.S. (2019). Bosniak Classification of Cystic Renal Masses, Version 2019: An Update Proposal and Needs Assessment. Radiology.

[3] Tateo V., Mollica V., Rizzo A., Santoni M., Massari F. (2023). Re: WHO Classification of Tumours, 5th Edition, Volume 8: Urinary and Male Genital Tumours. Eur. Urol..

[4] Mohanty S.K., Lobo A., Cheng L. (2023). The 2022 Revision of the World Health Organization Classification of Tumors of the Urinary System and Male Genital Organs: Advances and Challenges. Hum. Pathol..

[5] Spaliviero M., Herts B.R., Magi-Galluzzi C., Xu M., Desai M.M., Kaouk J.H., Tucker K., Steinberg A.P., Gill I.S. (2005). Laparoscopic Partial Nephrectomy for Cystic Masses. J. Urol..

[6] Chen S., Jin B., Xu L., Fu G., Meng H., Liu B., Li J., Xia D. (2014). Cystic Renal Cell Carcinoma: A Report of 67 Cases Including 4 Cases with Concurrent Renal Cell Carcinoma. BMC Urol..

[7] Akca O., Zargar H., Autorino R., Brandao L.F., Laydner H., Krishnan J., Samarasekera D., Li J., Haber G.-P., Stein R. (2014). Robotic Partial Nephrectomy for Cystic Renal Masses: A Comparative Analysis of a Matched-Paired Cohort. Urology.

[8] Novara G., La Falce S., Abaza R., Adshead J., Ahlawat R., Buffi N.M., Challacombe B., Dasgupta P., Moon D.A., Parekh D.J. (2016). Robot-Assisted Partial Nephrectomy in Cystic Tumours: Analysis of the Vattikuti Global Quality Initiative in Robotic Urologic Surgery (GQI-RUS) Database. BJU Int..

[9] Abdel Raheem A., Alatawi A., Soto I., Kim D.K., Kim L.H., Santok G.D., Lum T.G., Choi Y.D., Rha K.H. (2016). Robot-Assisted Partial Nephrectomy Confers Excellent Long-Term Outcomes for the Treatment of Complex Cystic Renal Tumors: Median Follow up of 58 Months. Int. J. Urol..

[10] Zennami K., Takahara K., Matsukiyo R., Nukaya T., Takenaka M., Fukaya K., Ichino M., Fukami N., Sasaki H., Kusaka M. (2021). Long-Term Functional and Oncologic Outcomes of Robot-Assisted Partial Nephrectomy for Cystic Renal Tumors: A Single-Center Retrospective Study. J. Endourol..

[11] Papalia R., Simone G., Ferriero M., Guaglianone S., Costantini M., Giannarelli D., Maini C.L., Forastiere E., Gallucci M. (2012). Laparoscopic and Robotic Partial Nephrectomy without Renal Ischaemia for Tumours Larger than 4 cm: Perioperative and Functional Outcomes. World J. Urol..

[12] Bertolo R., Autorino R., Simone G., Derweesh I., Garisto J.D., Minervini A., Eun D., Perdona S., Porter J., Rha K.H. (2018). Outcomes of Robot-Assisted Partial Nephrectomy for Clinical T2 Renal Tumors: A Multicenter Analysis (ROSULA Collaborative Group). Eur. Urol..

[13] Simone G., Tuderti G., Anceschi U., Ferriero M., Costantini M., Minisola F., Vallati G., Pizzi G., Guaglianone S., Misuraca L. (2019). “Ride the Green Light”: Indocyanine Green–Marked Off-Clamp Robotic Partial Nephrectomy for Totally Endophytic Renal Masses. Eur. Urol..

[14] Ferriero M., Brassetti A., Mastroianni R., Costantini M., Tuderti G., Anceschi U., Bove A.M., Misuraca L., Guaglianone S., Gallucci M. (2022). Off-Clamp Robot-Assisted Partial Nephrectomy for Purely Hilar Tumors: Technique, Perioperative, Oncologic and Functional Outcomes from a Single Center Series. Eur. J. Surg. Oncol..

[15] Tuderti G., Brassetti A., Mastroianni R., Misuraca L., Bove A., Anceschi U., Ferriero M., Guaglianone S., Gallucci M., Simone G. (2022). Expanding the Limits of Nephron-Sparing Surgery: Surgical Technique and Mid-Term Outcomes of Purely off-Clamp Robotic Partial Nephrectomy for Totally Endophytic Renal Tumors. Int. J. Urol..

[16] Kutikov A., Uzzo R.G. (2009). The R.E.N.A.L. Nephrometry Score: A Comprehensive Standardized System for Quantitating Renal Tumor Size, Location and Depth. J. Urol..

[17] Bertolo R., Simone G., Garisto J., Nakhoul G., Armanyous S., Agudelo J., Costantini M., Tuderti G., Gallucci M., Kaouk J. (2019). Off-Clamp vs on-Clamp Robotic Partial Nephrectomy: Perioperative, Functional and Oncological Outcomes from a Propensity-Score Matching between Two High-Volume Centers. Eur. J. Surg. Oncol..

[18] Ani I., Finelli A., Alibhai S.M.H., Timilshina N., Fleshner N., Abouassaly R. (2013). Prevalence and Impact on Survival of Positive Surgical Margins in Partial Nephrectomy for Renal Cell Carcinoma: A Population-Based Study. BJU Int..

[19] Pradere B., Peyronnet B., Delporte G., Manach Q., Khene Z.-E., Moulin M., Roumiguié M., Rizk J., Brichart N., Beauval J.-B. (2018). Intraoperative Cyst Rupture during Partial Nephrectomy for Cystic Renal Masses—Does It Increase the Risk of Recurrence?. J. Urol..

[20] Lee R.A., Uzzo R.G., Anaokar J., Thomas A., Wei S., Ristau B.T., McIntosh A., Lee M., Chen D.Y.T., Greenberg R.E. (2023). Pathological and Clinical Outcomes in a Large Surveillance and Intervention Cohort of Radiographically Cystic Renal Masses. J. Urol..

[21] Tse J.R., Shen L., Shen J., Yoon L., Kamaya A. (2021). Prevalence of Malignancy and Histopathological Association of Bosniak Classification, Version 2019 Class III and IV Cystic Renal Masses. J. Urol..

[22] Chandrasekar T., Ahmad A.E., Fadaak K., Jhaveri K., Bhatt J.R., Jewett M.A., Finelli A. (2018). Natural History of Complex Renal Cysts: Clinical Evidence Supporting Active Surveillance. J. Urol..

[23] Nouhaud F.X., Bernhard J.C., Bigot P., Khene Z.-E., Audenet F., Lang H., Bergerat S., Fraisse G., Grenier N., Cornelis F. (2018). Contemporary Assessment of the Correlation between Bosniak Classification and Histological Characteristics of Surgically Removed Atypical Renal Cysts (UroCCR-12 Study). World J. Urol..

[24] Yazdian Anari P., Zahergivar A., Gopal N., Chaurasia A., Lay N., Ball M.W., Turkbey B., Turkbey E., Jones E.C., Linehan W.M. (2024). Kidney Scoring Surveillance: Predictive Machine Learning Models for Clear Cell Renal Cell Carcinoma Growth Using MRI. Abdom. Radiol..

[25] Dana J., Lefebvre T.L., Savadjiev P., Bodard S., Gauvin S., Bhatnagar S.R., Forghani R., Hélénon O., Reinhold C. (2022). Malignancy Risk Stratification of Cystic Renal Lesions Based on a Contrast-Enhanced CT-Based Machine Learning Model and a Clinical Decision Algorithm. Eur. Radiol..

